# Transcriptional profiling of *Klebsiella pneumoniae* defines signatures for planktonic, sessile and biofilm-dispersed cells

**DOI:** 10.1186/s12864-016-2557-x

**Published:** 2016-03-15

**Authors:** Cyril Guilhen, Nicolas Charbonnel, Nicolas Parisot, Nathalie Gueguen, Agnès Iltis, Christiane Forestier, Damien Balestrino

**Affiliations:** Laboratoire Microorganismes: Génome Environnement, UMR CNRS 6023, Université d’Auvergne, Clermont Ferrand, F-63001 France; UMR 203 BF2I, Biologie Fonctionnelle Insectes et Interactions, INRA, INSA de Lyon, Université de Lyon, F-69621 Villeurbanne, France; Genostar, Montbonnot Saint Martin, F-38330 France

**Keywords:** *Klebsiella pneumoniae*, Biofilm, Dispersion, RNAseq, Transcriptional signatures

## Abstract

**Background:**

Surface-associated communities of bacteria, known as biofilms, play a critical role in the persistence and dissemination of bacteria in various environments. Biofilm development is a sequential dynamic process from an initial bacterial adhesion to a three-dimensional structure formation, and a subsequent bacterial dispersion. Transitions between these different modes of growth are governed by complex and partially known molecular pathways.

**Results:**

Using RNA-seq technology, our work provided an exhaustive overview of the transcriptomic behavior of the opportunistic pathogen *Klebsiella pneumoniae* derived from free-living, biofilm and biofilm-dispersed states. For each of these conditions, the combined use of Z-scores and principal component analysis provided a clear illustration of distinct expression profiles. In particular, biofilm-dispersed cells appeared as a unique stage in the bacteria lifecycle, different from both planktonic and sessile states. The K-means cluster analysis showed clusters of Coding DNA Sequences (CDS) and non-coding RNA (ncRNA) genes differentially transcribed between conditions. Most of them included dominant functional classes, emphasizing the transcriptional changes occurring in the course of *K. pneumoniae* lifestyle transitions. Furthermore, analysis of the whole transcriptome allowed the selection of an overall of 40 transcriptional signature genes for the five bacterial physiological states.

**Conclusions:**

This transcriptional study provides additional clues to understand the key molecular mechanisms involved in the transition between biofilm and the free-living lifestyles, which represents an important challenge to control both beneficial and harmful biofilm. Moreover, this exhaustive study identified physiological state specific transcriptomic reference dataset useful for the research community.

**Electronic supplementary material:**

The online version of this article (doi:10.1186/s12864-016-2557-x) contains supplementary material, which is available to authorized users.

## Background

Most bacteria can live in individual or community lifestyles. In the planktonic mode of growth, bacterial cells are free to move in suspension, whereas in the sessile state, they form surface-attached multicellular communities called biofilms. This dynamic heterogenic organization confers to its residents a powerful tolerance against stresses and facilitates symbiotic relationships between members of the communities [1, 2]. The transition between the planktonic and sessile modes of growth, as well as the biofilm development process are governed by environmental cues and the coordination of various molecular pathways linked notably to secondary messenger cyclic di-GMP and quorum sensing [3, 4]. Biofilm development progresses in three stages: i) bacterial attachment to a surface and formation of a monolayer biofilm, ii) maturation of the biofilm and emergence of a three-dimensional structure and iii) dispersion from mature biofilm.

The adhesion of planktonic cells to the surface is mostly driven by surface-exposed components like flagella, fimbriae and curli as observed in many bacteria [5]. Subsequent biofilm maturation is concomitant with the formation of an extracellular matrix composed of exopolysaccharides, DNA, lipids and proteins [6]. In *Pseudomonas aeruginosa* and *Escherichia coli,* exopolysaccharides and extracellular DNA also play a crucial role in the maturation process as the absence of these compounds severely impairs the formation of a three-dimensional structure [7].

The last step of the biofilm developmental process, dispersion from mature biofilm, constitutes an essential stage because of its crucial role in bacterial dissemination and colonization of new surfaces [8, 9]. It remains therefore unclear whether bacteria dispersed from biofilms represent or not a transition stage between biofilm and the planktonic lifestyle. Dispersion occurs either as individual cells or clumps [10], but the molecular mechanisms and effectors behind this process are still poorly documented [11]. Nevertheless, secreted effectors such as glycosidases in *Actinobacillus actinomycetemcomitans* [12], proteases in *Pseudomonas putida* [13], nucleases in *Haemophilus influenzae* [14] and biosurfactants in *Staphylococcus* [15, 16] are able to destabilize the biofilm structure and promote dispersion. Activation of prophages in *P. aeruginosa* and *Enterococcus faecalis* was also reported as inducing cell death inside microcolonies leading to biofilm dispersion [17, 18].

Despite the accumulation of data concerning the transcriptional profile of bacteria grown in different experimental models, there has been no documented overview of all states of biofilm development and dispersion. Transcriptomic approaches by microarray or RNA sequencing have attempted to address this issue in several bacterial species like *E. coli*, *P. aeruginosa* or *Acinetobacter baumannii*, and showed distinct expression profiles between sessile and planktonic stages. However, cells from dispersed biofilm were not included in these analyses [19–21].

The aim of this study was to identify the transcriptional landscape of the bacteria *Klebsiella pneumoniae* across different experimental growth states, i.e. planktonic, sessile, and spontaneously biofilm-detached bacteria. *K. pneumoniae* is an ubiquitous bacterium found both in nature and in clinical environments; the molecular mechanisms leading to biofilm formation have been previously investigated, mostly by punctual mutant analysis [22, 23]. In this work, comparison of the different whole transcriptomes obtained by RNA-seq showed that each lifestyle of *K. pneumoniae* was associated with a unique transcriptional behavior. The comprehensive overview provided by this study allowed the identification of specific transcriptional fingerprints for each state, including the biofilm-dispersed cells.

## Results

### Monitoring of biofilm development in a flow-cell model

**Fig. 1:**
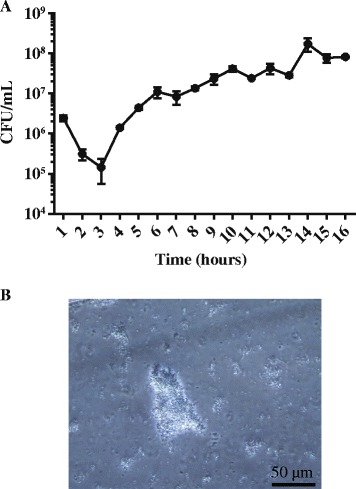
Number of viable bacteria in the flow-cell effluent. **a** The flow-cell with one chamber was inoculated with 10^8^ cells from an overnight culture of *K. pneumoniae* CH1034, and viable bacteria in the effluent were counted by plating every hour for 16 h. **b** Light microscopy observation of bacteria in the effluent after 12 h of incubation revealed the predominance of bacterial aggregates over individual cells

Monitoring of biofilm development by *K. pneumoniae* CH1034 in a flow-cell system with confocal microscopy showed initially the formation of microcolonies leading to the development of a flat structure after 7 h of incubation (T_7h_) (Additional file 1: Movie S1). At T_9h_, a three-dimensional structure was observable and potential detachment from this mature biofilm was then assessed. Bacteria in the flow-cell effluent were harvested throughout the experiment, and CFU determination of the resulting suspensions indicated that the number of viable cells decreased in the first 3 h of the experiment, from 5.10^6^ CFU/mL (T_1h_) to 1.10^5^ (T_3h_), owing probably to the elimination of planktonic non-adhering cells (Fig. 1a). Observation of the harvested samples by optical microscopy revealed mainly individual bacteria (data not shown). From T_3h_ to T_6h_, the number of viable bacteria in the effluent increased rapidly and then progressively in the following 10 h (T_6h_ to T_16h_) (Fig. 1a). Microscopic observations revealed a progressive appearance of bacterial aggregates in the effluent, which predominated over individual cells after 12 h of incubation (Fig. 1b).

### Planktonic, sessile, and biofilm-detached bacteria presented distinct transcriptional profiles

**Fig. 2:**
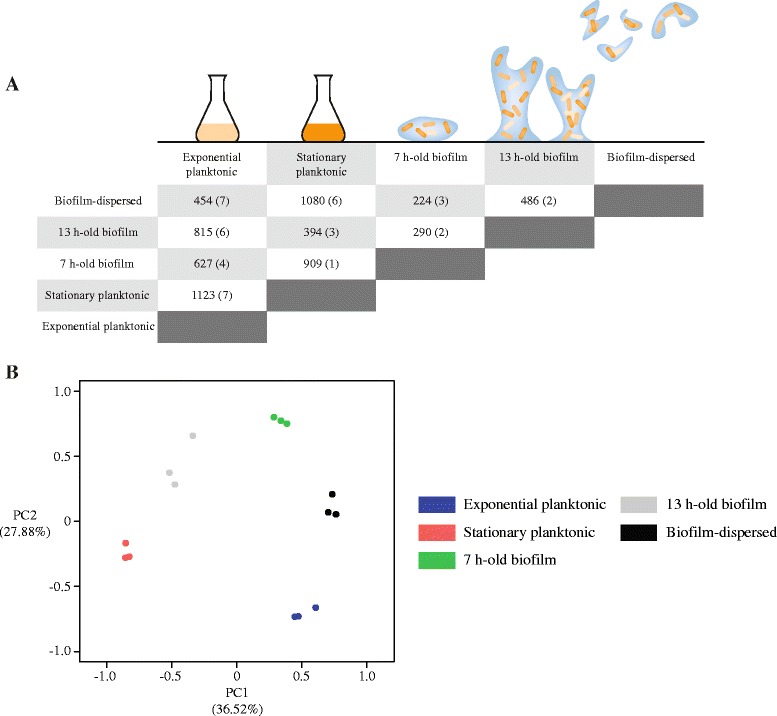
Comparison of the *K. pneumoniae* CH1034 gene expression levels across the different conditions. **a** The expression levels of the 5 146 CDS and the 44 ncRNA genes of the *K. pneumoniae* CH1034 genome were compared in each of the 10 possible pairs of conditions. The number of differentially expressed (∣fold-change∣ > 5 and adjusted *P*-value < 0.01) CDS and the number of ncRNA genes, shown in parentheses, are indicated for each comparison. **b** Principal component analysis (PCA) of gene expression in the five growth conditions. PCA was performed with Z-score values of the 2 052 CDS and 19 ncRNA genes differentially expressed (∣fold-change∣ > 5 and adjusted *P*-value < 0.01) in at least one of the 10 possible pairs of conditions. Z-score values were calculated with absolute expression values normalized by the DESeq package, and were used as a matrix to perform a PCA with package FactoMineR of R/Bioconductor. Each dot indicates a biological replicate. The lists of these 2 052 CDS and the 19 ncRNA genes are provided respectively in Additional file 2: Table S1 and Additional file 3: Table S2

Transcriptional analysis was performed with sessile bacterial cells collected before and after the formation of a three-dimensional structure, at T_7h_ and T_13h_, respectively. Detached cells isolated in the flow-cell effluent (T_12h_-T_13h_), exponential and stationary growing planktonic cells were also included. RNAseq analysis indicated that 2 052 of the 5 146 CDS of *K. pneumoniae*, as well as 19 of the 44 annotated ncRNA genes (excluding tRNA and rRNA genes), were differentially expressed in at least one of the ten possible pairs of conditions (∣fold-change∣ > 5 and adjusted *P*-value < 0.01) (Fig. 2a), with fold-changes ranging from −2 780 to 2 182 (Additional file 2: Table S1; Additional file 3: Table S2). To validate the RNA-seq efficiency, 20 genes differentially expressed between the 13 h-old biofilm bacteria and the bacteria collected in the effluent (10 genes overexpressed and 10 genes under-expressed; *P*-value < 0.01) were randomly selected. Their relative expression levels were determined by RT-qPCR with total RNA extracted from cells harvested in two conditions: bacteria in the effluent and 13 h-old biofilm. Results indicated a high correlation between RNAseq and RT-qPCR data (*r* = 0.97; *P*-value < 0.0001; Pearson’s correlation test) (Additional file 4: Figure S1).

PCA performed with Z-score values of the 2 052 CDS and 19 ncRNA genes indicated that the first principal component (PC1) accounted for 36.52 % and the second principal component (PC2) for 27.88 % of the total variation in the dataset (Fig. 2b). A plot of these Z-score values against a heatmap (Additional file 5: Figure S2) and the proximity of points in the PCA (Fig. 2b) demonstrated the high reproducibility of the data among the replicates. In addition, such analysis clearly indicated that all bacterial states (planktonic, sessile and bacteria in the effluent) exhibited specific transcriptional profiles (Fig. 2b and Additional file 5: Figure S2), and suggests that bacterial cells in the effluent are not pieces of biofilm mechanically detached from the biomass. Hereafter they will be referred to as biofilm-dispersed cells.

The transcriptome of the biofilm-dispersed cells presented only 224 CDS and 3 ncRNA genes differentially expressed (∣fold-change∣ > 5 and adjusted *P*-value < 0.01) when compared with those of the 7 h-old biofilm state. In contrast, 454 CDS and 7 ncRNA genes, 486 CDS and 2 ncRNA genes, and 1 080 CDS and 6 ncRNA genes were differentially expressed (∣fold-change∣ > 5 and adjusted *P*-value < 0.01) when compared with those of exponential planktonic state, 13 h-old biofilm and stationary planktonic state, respectively (Fig. 2a). Hence, biofilm-dispersed cells harbored a distinct transcriptional profile, which was closer to that of bacteria from 7 h-old biofilm than to that of 13 h-old biofilm and planktonic cells.

### Gene functional classification of *K. pneumoniae* lifestyles through K-means clustering

**Fig. 3:**
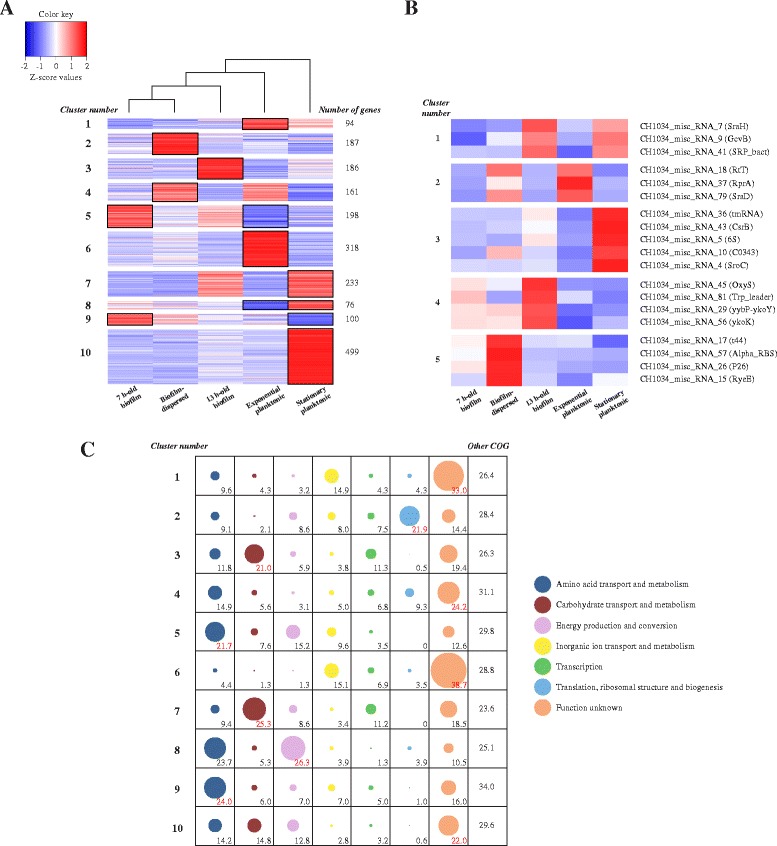
K-means clustering of the Z-score values of each differentially expressed CDS and ncRNA genes in the five different growth conditions, and Clusters of Orthologous Group (COG) affiliation of the CDS of each K-means cluster. **a** Heatmap depicting the K-means clustering of the 2 052 differentially expressed CDS in 10 clusters with column hierarchical clustering. The average Z-scores of the 10 clusters was calculated for each condition, and the 13 clusters presenting an average Z-score value > 1 or < −1 were framed. Blue and red clusters gathered genes under- or overexpressed compared to the mean, respectively (**b**) K-means clustering of the 19 differentially expressed ncRNA genes in 5 clusters. Locus tag of each ncRNA gene, and its respective annotation in parentheses, are indicated. Blue and red clusters gathered genes under- or overexpressed compared to the mean, respectively (**c**) Clusters of Orthologous Group (COG) affiliation of the CDS of each K-means cluster. Only COG categories containing more than 10 % of the CDS of one cluster are presented. The circle size is proportional to the percentage of CDS (indicated by numbers) affiliated to a COG category for one given cluster group. Percentages in red correspond to the major part of each cluster. COG categories not presented are grouped in the “other COG” category. An exhaustive view of the CDS composition of each cluster and their COG affiliation is provided in Additional file 2: Table S1 and in Additional file 6: Figure S3

K-means clustering was then used to visualize the distribution of the expression levels of the 2 052 CDS and the 19 ncRNA genes differentially expressed (∣fold-change∣ > 5 and adjusted *P*-value < 0.01) in the different conditions (Fig. 3a and b). Owing to the high reproducibility of data, Z-score values were able to be calculated with average values from normalized DEseq counts. This clustering indicated that the clearest representation was obtained with K = 10 for the CDS analysis and K = 5 for the ncRNA genes analysis, and showed different transcriptomic profiles between conditions. In Fig. 3a, with clusters ranging from 76 to 499 CDS for clusters 8 and 10 respectively, column clustering confirmed that dispersed cells were transcriptionally closer to 7 h-old biofilm cells than to those in all the other conditions, whereas stationary phase cells were the most different group of this dataset.

In order to highlight groups of genes highly overexpressed or under-expressed in a specific condition, the mean of the Z-scores in each cluster in the Fig. 3a was calculated for each condition. Only the Z-score groups presenting a mean value > 1 or < −1, named overexpressed boxes and under-expressed boxes, respectively (framed in Fig. 3a), were considered thereafter. All clusters presented only one overexpressed box, but clusters 5, 8 and 9 also presented one under-expressed box (Fig. 3a).

Analysis of the potential function of protein-coding genes in the under-expressed and overexpressed boxes by the Clusters of Orthologous Groups (COG) classification is represented in Fig. 3c and Additional file 6: Figure S3. A large number of genes were poorly characterized and therefore categorized in the “unknown function” class. Exponential planktonic cells exhibited two overexpressed boxes (clusters 1 and 6) (Fig. 3a), containing CDS mainly involved in inorganic ion transport and metabolism (14.9 and 15.1 % of the genes present in clusters 1 and 6, respectively) (Fig. 3c and Table 1). In parallel, two under-expressed boxes (clusters 5 and 8) were identified in the exponential planktonic condition. They contained mainly CDS involved in amino acid transport and metabolism, and energy production and conversion, as defined by the COG classification. Stationary planktonic cells exhibited three overexpressed boxes (clusters 7, 8 and 10) that contained CDS mostly implied in energy production and conversion, and in amino acid and carbohydrate transport and metabolism. The 7 h-old biofilm cells exhibited two overexpressed boxes (clusters 5 and 9) (Fig. 3a), which contained CDS chiefly involved in amino acid transport and metabolism (21.7 and 24 % of the genes present in clusters 5 and 9, respectively) (Fig. 3c and Table 1). The 13 h-old biofilm cells exhibited one overexpressed box (cluster 3), with CDS chiefly involved in carbohydrate transport and metabolism (21 % of the genes present in cluster 3). Finally, dispersed cells exhibited two overexpressed boxes (clusters 2 and 4), containing CDS chiefly involved in translation, ribosomal structure and biogenesis (21.9 and 9.3 % of the genes present in clusters 2 and 4, respectively).

**Table 1:** Summary of the COG affiliation for the under-expressed and overexpressed boxes in each condition

Condition	Cluster containing under-expressed box	Cluster containing overexpressed box	COG affiliation ^a^	
Exponential planktonic	5 and 8		Amino acid transport and metabolism - Energy production and conversion	
		1 and 6	Inorganic ion transport and metabolism - Function unknown	
Stationary planktonic	9		Amino acid transport and metabolism - Function unknown	
		7 and 10	Carbohydrate transport and metabolism - Function unknown	
		8	Energy production and conversion - Amino acid transport and metabolism	
7 h-old biofilm		5	Energy production and conversion	Amino acid transport and metabolism
		9	Function unknown	
13 h-old biofilm		3	Carbohydrate transport and metabolism - Function unknown	
Biofilm-dispersed		2	Translation, ribosomal structure and biogenesis	Function unknown
		4	Amino acid transport and metabolism	

^a^Only the two most representative COG affiliations of each cluster were displayed

### Identification of a set of signature genes for each condition

**Fig. 4:**
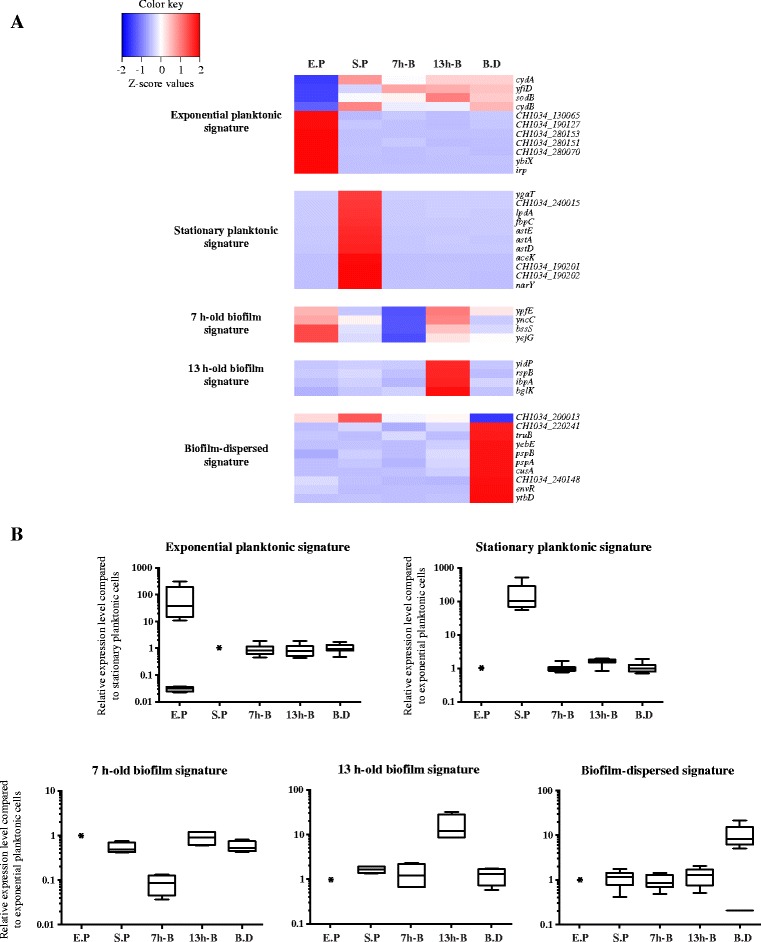
Relative expression levels of the different signature genes in their respective condition. **a** Z-score values of the selected signature genes were calculated using average values from normalized DESeq counts and plotted against a heatmap. **b** Boxplot of the relative expression levels of each signature gene were compared with those in exponential planktonic condition, except for the exponential planktonic condition, which was compared with the stationary planktonic condition; * represents the normalized expression value for the reference condition. (E.P: exponential planktonic; S.P: stationary planktonic; 7 h-B: 7 h-old biofilm; 13 h-B: 13 h-old biofilm; B.D: biofilm-dispersed)

Since clustering suggested the existence of specific signature genes for each condition, different stringent threshold fold-changes were applied to extract the most relevant transcriptional signature genes, up- or down-regulated, for each condition (Additional file 7: Figure S4). Forty signature CDS were identified, 11 associated with the exponential and the stationary planktonic states, 4 with the 7 h-old and the 13 h-old biofilm cells, and 10 with biofilm dispersal (Table 2). In the stationary planktonic and 13 h-old biofilm conditions, all signature CDS were upregulated, and in the 7 h-old biofilm condition, all were down-regulated, whereas exponential planktonic cells and biofilm-dispersed cells displayed both up- and down-regulated signature CDS (Table 2 and Fig. 4). The Z-score values of these 40 CDS plotted against a heatmap (Fig. 4a) and their relative expression level (Fig. 4b) confirmed their signature singularity. Putative functions of these protein encoding signatures CDS are listed in Table 2 and concern mainly transport, transcriptional regulation and metabolic pathways.

**Table 2:** List of the 40 selected signature genes with their respective annotation and their DESeq normalized counts for each experimental condition

Signature condition	Locus Tag	Name	Annotation	DESeq normalized expression (baseMeans^a^)					
				Exponential planktonic	Stationary planktonic	7 h-old biofilm	13 h-old biofilm	Biofilm-dispersed	K-means cluster affiliation
Exponential planktonic	CH1034_160111	*cydA*	cytochrome d terminal oxidase, polypeptide subunit I	1265.85	39855.85	25832.68	32264.31	32480.00	8
	CH1034_270098	*yfiD*	Autonomous glycyl radical cofactor	751.58	25251.54	47481.65	46582.09	42762.11	5
	CH1034_230111	*sodB*	superoxide dismutase, Fe	556.16	14965.09	16289.86	25654.59	19597.48	3
	CH1034_160112	*cydB*	cytochrome d terminal oxidase, subunit II	538.42	25053.85	12872.09	13297.53	20932.48	8
	CH1034_130065		DNA polymerase	10233.37	426.38	947.64	642.98	932.75	6
	CH1034_190127		Short-chain dehydrogenase/reductase SDR	2269.29	139.01	107.51	59.95	139.54	6
	CH1034_280153		TonB-dependent receptor	77058.51	476.51	469.04	460.15	548.80	6
	CH1034_280151		conserved protein of unknown function	3482.87	166.75	226.75	109.19	139.51	6
	CH1034_280070		GntR-family bacterial regulatory protein	3552.42	331.98	281.55	332.91	258.67	6
	CH1034_190125	*ybiX*	Fe(II)-dependant oxygenase	5077.73	93.32	59.63	72.73	126.67	6
	CH1034_250006	*irp*	High-molecular-weight protein 2	260772.06	836.60	383.18	377.66	390.89	6
Stationary planktonic	CH1034_130044	*ygaT*	Carbon starvation induced protein	4.91	1192.69	3.93	8.99	5.23	10
	CH1034_240015		Ferric iron ABC transporter, permease protein	30.36	8565.31	25.54	48.18	37.72	10
	CH1034_130056	*lpdA*	Dihydrolipoyl dehydrogenase	138.96	17648.19	131.68	244.32	123.71	10
	CH1034_240014	*fbpC*	Fe(3+) ions import ATP-binding protein FbpC	34.43	10241.53	38.18	56.29	33.07	10
	CH1034_190101	*astE*	succinylglutamate desuccinylase	24.92	2503.60	20.16	20.31	28.51	10
	CH1034_190098	*astA*	arginine succinyltransferase	68.46	3714.09	49.29	102.14	48.99	10
	CH1034_190322	*astD*	succinylglutamate semialdehyde dehydrogenase	10.78	788.81	10.46	20.76	19.92	10
	CH1034_60005	*aceK*	isocitrate dehydrogenase kinase/phosphatase	384.73	194088.29	347.77	342.45	696.83	10
	CH1034_190201		Glycoside hydrolase	150.45	14489.44	140.46	218.27	116.28	10
	CH1034_190202		Histidine kinase	253.69	17137.61	266.30	405.62	173.46	10
	CH1034_220300	*narY*	Nitrate reductase 2 subunit beta	168.04	10624.15	267.91	294.28	144.35	10
7 h-old biofilm	CH1034_260051	*ypfE*	Carboxysome structural protein	21.40	11.37	2.19	26.02	17.63	3
	CH1034_220103	*yncC*	MFS transporter	634.15	478.67	84.96	713.34	340.06	1
	CH1034_180150	*bssS*	biofilm regulator	24731.65	10960.85	1747.59	16844.60	10452.60	1
	CH1034_250228	*yejG*	hypothetical protein	11084.19	4607.89	407.32	6588.61	5798.71	1
13 h-old biofilm	CH1034_220106	*yidP*	Transcriptional regulator, GntR family protein	109.08	208.43	197.27	3617.98	125.67	3
	CH1034_300308	*rspB*	putative oxidoreductase, Zn-dependent and NAD(P)-binding	180.70	248.45	122.42	1590.79	106.96	3
	CH1034_10036	*ibpA*	heat shock chaperone	3204.61	4686.30	2252.81	29872.19	4924.76	3
	CH1034_270020	*bglK*	Beta-glucoside kinase	177.63	350.06	423.50	2757.26	316.70	3
Biofilm-dispersed	CH1034_200013		conserved protein of unknown function	5547.43	8030.40	4651.95	5015.35	1035.49	10
	CH1034_220241		Transcriptional regulator, LysR family	700.27	991.71	507.89	989.76	4255.17	2
	CH1034_300259	*truB*	tRNA pseudouridine synthase B	1963.34	1548.32	2797.65	1589.07	15569.23	2
	CH1034_240296	*yebE*	conserved hypothetical protein; putative inner membrane protein	385.81	448.95	315.41	608.81	5089.16	2
	CH1034_190182	*pspB*	phage-shock-protein B	147.34	256.45	198.93	298.93	1192.17	2
	CH1034_190181	*pspA*	phage-shock-protein A	855.89	991.23	730.55	1195.88	5254.86	2
	CH1034_100015	*cusA*	copper/silver efflux system, membrane component	87.92	95.96	110.78	176.25	1854.58	2
	CH1034_240148		multidrug DMT transporter permease	83.27	54.21	43.60	41.28	417.64	2
	CH1034_330036	*envR*	DNA-binding transcriptional regulator	27.10	11.16	12.72	14.23	232.73	2
	CH1034_130003	*ytbD*	MFS sugar transporter	50.42	60.24	47.49	59.31	846.18	2

^a^BaseMeans are the DESeq normalized values from the averaged triplicates of a condition

## Discussion

In the present study, the transcriptional changes occurring in the course of *K. pneumoniae* biofilm formation and biofilm-detachment were characterized by RNAseq. To date, the few data available on biofilm dispersion were obtained with artificial dispersion signals such as c-di-GMP depletion [24, 25]. In contrast, we investigated spontaneous biofilm-detached cells. Results indicated that each of the tested *K. pneumoniae* lifestyles, i.e. planktonic (exponential and stationary phases), sessile (7 h-old and 13 h-old biofilms) and biofilm-dispersed cells, exhibit unique and specific transcriptional profiles. The comprehensive overview presented in this study allowed the analysis of the transcriptional fate of all *K. pneumoniae* genes in different bacteria lifestyles.

The stationary planktonic mode of growth displayed the most particular pattern with 499 genes highly overexpressed in the K-means cluster 10. Entry in the stationary phase is the result of nutrient starvation and in consequence bacteria modulate the expression level of a considerable number of genes, many of them being under the control of the stationary-phase sigma S factor (σ^S^) [26]. On the basis of a study referencing the 100 most RpoS-dependent genes in stationary phase of a pathogenic *E. coli* strain [27], 54 of the 82 genes present in the *K. pneumoniae* genome were found in the K-means cluster 10, including 4 transcriptional signature genes of the stationary phase (*ygaT* (also named *csiD*), *astA*, *astD* and *astE*). Overall, the predominance of σ^S^-dependent genes upregulated in stationary phase cells emphasized the accuracy of our data. With 1 123 differentially expressed genes, stationary planktonic cells were transcriptionally different from exponential planktonic cells (Fig. 2a), as reported elsewhere [20]. Interestingly, three genes belonging to the same operon, *cydA*, *cydB* and *ybgT* (also named *cydX*), were under-expressed in exponential planktonic cells, and two of them, *cydA* and *cydB*, were selected as signature genes. In *E. coli*, the *cyd* operon encodes the three subunits of the cytochrome *bd* oxygen reductase complex, whose expression is induced under stressful growth conditions [28, 29]. The non-nutrient-limited early planktonic mode of growth explains the under-expression of this complex but also, more generally, the under-expression of pathways involved in energy production and conversion (see COG affiliation of clusters 5 and 8 in Fig. 3c and Table 1).

The response regulator CsgD, a master transcriptional regulator in biofilm formation, functions by assisting bacterial cells in transitioning from the planktonic stage to the multicellular state through the activation of expression of biofilm-linked genes [30, 31]. Accordingly, CsgD encoding gene was 25.0-fold overexpressed in 7 h-old biofilm compared to stationary planktonic growing cells, although its expression did not significantly change between the two sessile conditions. However, transcriptomic profiles of the 7 h-old and 13 h-old biofilm cells contained 290 differentially expressed CDS (∣fold-change∣ > 5 and adjusted *P*-value < 0.01) (Fig. 2a), which shows an evolution of the biofilm structure between these two time points and validates our experimental model. These findings are in agreement with those of previous studies showing distinct transcriptomic profiles in developing and confluent biofilm states [20, 21]. Genes of clusters 5 and 9 were specifically overexpressed in 7 h-old biofilm, showing that amino acid transport and metabolism (see COG affiliation in Table 1) is an essential process during the biofilm growth, as observed previously [32–34]. The *bssS* gene, encoding a biofilm regulator whose inactivation leads to an increase in both the biomass and thickness of biofilm in *E. coli* [35], was an under-expressed signature gene of the 7 h-old biofilm condition. In a more mature biofilm, 13 h-old biofilm, the overexpression of genes involved in carbohydrate transport and metabolism (cluster 3; Table 1) reflect the importance of sugar in the formation of the extracellular matrix, a crucial component for biofilm maturation [6]. The *ibpA* gene was identified among the overexpressed signature genes of the 13 h-old biofilm condition, and encodes a heat shock protein whose overexpression is crucial in *E. coli* during biofilm growth [36].

The transcriptional pattern of bacteria harvested in the effluent was also specific. Surprisingly, according to K-means column clustering and the number of differentially expressed genes in the different conditions, biofilm-dispersed cells were transcriptionally closer to the 7 h-old biofilm cells than to the planktonic cells. Our results showed that dispersed cells represent a distinct stage in the bacteria lifecycle, different from both the planktonic and the biofilm states. Environmental pressure could then influence the fate of these cells converting them either into planktonic cells as suggested by Chua et al. [24] or into new biofilm structures.

Because spontaneously dispersed-cells were analyzed, the question of any potential input signal triggering the dispersion process was assessed. Quorum-sensing signaling is important for the proper regulation of biofilm development in several species, including *K. pneumoniae* [7, 37]. In our study, the operons *lsrACDBFG* and *lsrRK* encoding the regulatory network for AI-2 did present a strong up-regulation between 7 h-old biofilm and 13 h-old biofilm conditions. Interestingly, these genes were significantly under-expressed in dispersed cells compared to 13 h-old biofilm cells. Since the *lsrACDBFG* operon is transcriptionally regulated by both the LsrR repressor and the phosphoenolpyruvate phosphotransferase system (PTS), its expression could depend on the availability of certain substrates and the global metabolic status of the cell [38]. In this way, our data suggested that *lsr* gene modulation and the subsequent down-regulation of the biofilm-linked genes trigger the dispersal process. Biofilm dispersal involving high concentrations of extracellular AI-2 was recently reported in *E. faecalis* and has been shown to be associated with phages release by sessile cells [18]. A biofilm dispersal mechanism mediated by filamentous prophage-induced cell death has also been reported in *P. aeruginosa* [17, 39]. In our study, among the 10 transcriptional signature genes of biofilm-dispersed cells, *pspA* and *pspB*, encoding phage shock proteins A and B, were overexpressed (Fig. 4 and Table 2). Since the phage-shock protein A was overproduced in *E. coli* during filamentous phage infection [40, 41], it is tempting to hypothesize that the overexpression of the *pspABCDE* operon in *K. pneumoniae* dispersed cells is the consequence of bacteriophage activation, which leads to local cell death and therefore biofilm dispersal.

Since c-di-GMP depletion plays an important role in the dispersal from mature biofilms in many species [4, 42], we analyzed the expression of genes encoding proteins containing GGDEF (diguanylate cyclases) and EAL domains (phosphodiesterases), which catalyze the formation and the degradation of c-di-GMP, respectively. Two diguanylate cyclases encoding genes (*CH1034_220201* and *CH1034_50012*) and one phosphodiesterase encoding gene (*CH1034_280331* or *mrkJ*) were, respectively, under- and overexpressed in dispersed cells compared to 13 h-old biofilm cells. The phosphodiesterase activity of MrkJ in *K. pneumoniae* is an important factor in the regulation of type 3 fimbriae expression, which mediates the formation and disassembly of the biofilm [43]. Among the other candidates potentially involved in the dispersal process, some degrading matrix enzyme-encoding genes were overexpressed in dispersed cells compared to 13 h-old biofilm, such as the protease-encoding gene *ycbZ*, the glucosidase-encoding gene *malZ* and the nucleases encoding genes *endA*, *rnhB*, *nth*, and *yihG*. Interestingly, genes involved in the SOS response (*dinB*, *dinF, dinG*, *dinI*, *sulA*, *recA* and *recX*) were also overexpressed in dispersed cells compared to 13 h-biofilm cells, suggesting a role of the stress response in biofilm dispersal. Although SOS stress response had not been directly related to biofilm dispersion, several studies reported the impact of nitrosative and nutrient stress on biofilm dispersal [13, 44]. Regarding the transcriptional status of the biofilm-dispersed cells, 21.9 and 9.3 % of the overexpressed genes in the K-means clusters 2 and 4, respectively, were categorized in the “translation, ribosomal structure and biogenesis” COG group (Fig. 3c). Dispersal probably requires high metabolic activity, even higher than that of the exponential planktonic cells. Indeed, only 4.3 and 3.5 % of the genes categorized in the K-means clusters 1 and 6, respectively (and therefore overexpressed in exponential planktonic condition), also belong to this COG group (Fig. 3c). However, ribosomal proteins could act not only in protein synthesis but also as regulators of the biofilm life cycle, as recently shown with the ribosomal proteins S11 (*rpsK*) and S21 (*rpsU*) in *Bacillus subtilis* [45]. Another interesting feature of dispersed cells was the overexpression of *cusA* (Fig. 4 and Table 2), a member of the *cusCFBA* operon encoding a cation tripartite efflux pump involved in the detoxification of cooper and silver ions in the periplasm of *E. coli* [46]. Two *cusCFBA* operons are present in the *K. pneumoniae* CH1034 genome and both were specifically overexpressed in dispersed cells (Additional file 2: Table S1). Because efflux systems have a major role in host colonization [47], we can therefore hypothesize that *K. pneumoniae* dispersed cells display specific phenotypes with high adaptive ability to colonize a new hostile environment. This hypothesis is reinforced by the fact that RyeE and t44, ncRNA genes, were overexpressed in dispersed cells (cluster 5, Fig. 3b); RyeE is upregulated in *Yersinia pestis* during lung infection [48] and the t44 expression level increases during initial invasion of fibroblast by *Salmonella* serovar Typhimurium [49].

## Conclusions

Several works have already described the transcriptomic profile of biofilm cells [19–21] but none of them ever considered the overall cycle of bacterial life. The present study provides an exhaustive view of the transcriptional behavior of *K. pneumoniae* in the course of planktonic, biofilm formation and dispersion steps. By structuring data in clusters, we achieved a clear illustration of the specific expression profiles and functions, and identified signature genes as potential biomarkers of the different bacterial states. Further research on the genes evidenced in our work will provide a better understanding of the molecular mechanisms involved in the transition between planktonic, sessile and dispersed states.

## Methods

### Bacterial strains and culture conditions

*K. pneumoniae* CH1034 was grown in Lysogeny broth (LB) or in 0.4 % glucose M63B1 minimal medium (M63B1) at 37 °C with shaking and stored at −80 °C in LB broth containing 15 % glycerol. For subsequent RNA extraction, planktonic bacteria were cultured at 37 °C in M63B1 broth under aerobic conditions and harvested at OD_620_ = 0.25 (exponential phase) or after overnight growth (stationary phase).

### GFP-tagged strain construction

The *K. pneumoniae* CH1034 GFP-tagged strain was constructed after replacement of the SHV-1 β-lactamase-encoding gene (chromosomal ampicillin resistance) by the selectable *aadA7*-*gfp*mut3 cassette. Briefly, the *aadA7*-*gfp*mut3 cassette flanked by 60-bp fragments, which correspond to the encoding upstream and downstream regions of *shv*, was generated using pKD4 plasmid as template, primers shv-GFP-Fw and shv-GFP-Rv and Phusion high-Fidelity DNA polymerase (Thermo Fisher Scientific, Waltham, Massachusetts, USA) according to the manufacturers’ recommendations. Primers were designed on the basis of information about the *K. pneumoniae* CH1034 genome sequence previously deposited in the ENA/EMBL-EBI database under the accession number: PRJEB9899 [50]. The PCR fragment was then transformed by electroporation into the 0.4 % arabinose-induced *K. pneumoniae* CH1034 strain harboring the pKOBEG199, which contains the lambda-red proteins encoding genes under the control of a promoter induced by l-arabinose [22]. The *K. pneumoniae* CH1034 GFP-tagged strain, named *K. pneumoniae* CH1034-*gfp*, was selected onto LB agar containing spectinomycin (70 μg/mL), and the loss of the pKOBEG199 plasmid was then checked by plating onto LB agar containing tetracycline (35 μg/mL).

### Flow-cell experiments

Two types of flow-cell devices were used in this study, a flow-cell with three individual chambers (dimension: 35 x 1 x 5 mm; 175 mm^3^) to monitor biofilm development by confocal laser scanning microscopy, and a flow-cell with one chamber (dimension: 54 x 19 x 6 mm; 6156 mm^3^) for i) quantification and microscopic observations of the bacteria detached from biofilm, and ii) bacterial recovery for RNA-extraction. On both flow-cells, a glass cover slip ensuring a surface for biofilm development was glued with silicon glue (3 M, Saint Paul, Minnesota, USA). All components of the flow-cell system, including tubing, bubble traps, medium/waste bottles and flow-cell, were assembled as described previously [51]. Before experiments, the system was sterilized by pumping 10 % (wt/vol) hypochlorite sodium for 1 h and then ethanol 100 % (vol/vol) for 15 min. Thereafter, the system was rinsed with M63B1 medium overnight at 37 °C. The inoculum composed of an overnight culture of *K. pneumoniae* CH1034 in M63B1 (4.10^6^ and 10^8^ cells for the three- and one-chamber flow-cells, respectively) was injected with a syringe into each compartment of the flow-cells. After 1 h of incubation at 37 °C without flow to allow bacterial adhesion, M63B1 medium was pumped at a constant rate of 0.08 mL/min (three-chamber flow-cell) or 0.9 mL/min (one-chamber flow-cell) through the devices.

Biofilm development was monitored in real time with an SP5 confocal laser microscope (Leica, Wetzlar, Germany) and a x40 oil objective. Images were processed with IMARIS software (Bitplane, Belfast, United Kingdom). Bacteria present in the effluent of the one-chamber flow-cell were observed with the Leica DM1000 optical microscope (Leica) and the Leica DFC295 camera (Leica). To quantify bacteria detached from the biofilm, viable bacteria present in the effluent were counted every hour for 16 h by serial dilution and plating on LB agar. For RNA extraction, biofilms developed on glass slide were recovered after 7 h or 13 h of incubation, and bacteria detached from the biofilm were recovered in the flow-cell effluent for 1 h after 12 h of incubation.

### RNA-seq and RT-qPCR

For RNA-sequencing, total RNA was extracted from biological triplicate of planktonic, sessile or biofilm-detached bacteria prepared as described below. To avoid transcriptional changes and RNA degradation, all bacteria sampled were prepared in RNA*later*® solution (Thermo Fisher Scientific) and then stored at 4 °C until RNA extraction. For exponential phase and stationary phase planktonic samples, an equivalent of 10^10^ CFU were pelleted by centrifugation at 6 000 g for 5 min at 4 °C, and pellets were resuspended in 2 mL of RNA*later*® solution. To prepare the 7 h-old biofilm and the 13 h-old biofilm samples, biofilms developed on the glass slide of the flow-cell after the defined incubation period were scrapped in 1 mL of RNA*later*® solution. In order to recover biofilm-detached bacteria, effluent of the flow-cells was directly collected in RNA*later*® solution. After 1 h of collection, samples were centrifuged at 6 000 g for 5 min at 4 °C, and pellets were resuspended in 2 mL of RNA*later*® solution. Before RNA extraction, bacteria were washed twice with 1X PBS. Total RNA was extracted according to the method described by Toledo-Arana et al. [52]. Briefly, bacteria were mechanically lysed with the PreCellys 24 system (Bertin Technologies, Montigny le Bretonneux, France) at speed of 6 500 rpm for two consecutive cycles of 30 s. After acid phenol (Thermo Fisher Scientific) and TRIzol® (Thermo Fisher Scientific) extraction, total RNA was precipitated with isopropanol and treated with 10 units of TURBO DNase (Thermo Fisher Scientific). After a second phenol-chloroform extraction and ethanol precipitation, RNA pellets were suspended in DEPC-treated water. RNA concentrations were quantified with the Qubit system (Thermo Fisher Scientific) and RNA qualities were determined with Agilent RNA 6000 Pico chip (Agilent Technologies, Santa Clara, California, USA). Ribosomal RNA (rRNA) were removed from each total RNA sample with the Ribo-Zero Magnetic Kit (Bacteria) (Epicentre Biotechnologies, Madison, Wisconsin, USA), and rRNA-depleted samples were checked with Agilent RNA 6000 Pico chip. RNA-sequencing (RNA-seq) was conducted by MGX GenomiX (Montpellier, France). Libraries were produced by the Illumina TruSeq Stranded messenger RNA Sample Preparation Kit, and sequenced with the HiSeq 2000 system (Illumina, San Diego, California, USA) with a single-end protocol and read lengths of 50-bp. Short reads were mapped against the genome of *K. pneumoniae* CH1034 with the Burrows-Wheeler Alignment-backtrack mapper (version 0.7.12-r1039) [53], which allows a maximum of two mismatches within the first 32-bp. Counting was performed with the software HTSeq-count using the union mode. As data come from a strand-specific assay, the read has to be mapped to the reverse strand of the gene. Analysis of the reads mapped to intergenic regions confirmed the overall quality of the genome annotation and therefore strengthen the choice to focus on CDS and ncRNA features. Differentially expressed CDS and ncRNA genes between any pair comparisons of the five groups were determined by a negative binomial test with the DESeq package of R/Bioconductor. Transcripts were considered as differentially expressed using the following criteria: *P*-value < 0.01 and ∣fold-change∣ > 5. Transcriptome sequencing data were deposited in the Gene Expression Omnibus (GEO) database under the GEO accession number: GSE71754.

Reverse transcription was performed with 500 ng of total RNA prepared as described above, and the absence of DNA contamination was verified by qPCRs performed with primer pair RT-cpxR-Fw/RT-cpxR-Rv and the SsoAdvanced SYBR® Green Supermix (Bio-Rad, Hercules, California, USA) according to the manufacturer’s recommendations. cDNA were prepared with the iScript cDNA Synthesis kit (Bio-Rad) under the following conditions: 5 min at 25 °C, 30 min at 42 °C and 5 min at 85 °C. qPCRs were carried out in the CFX96 Real Time System (Bio-Rad) with the SsoAdvanced SYBR® Green Supermix (Bio-Rad) under the following conditions: initial denaturation at 95 °C for 30 s, and 40 cycles of 5 s at 95 °C and 20 s at 59 °C. qPCRs were performed in 10 μL total volume per well containing 1X SYBR® Green, 625 nM of each gene-specific primer and 2 μL of 20X diluted cDNA. Primers were designed on the basis of *K. pneumoniae* CH1034 genome sequence information [50] and are listed in Additional file 8: Table S3. Melting curve analysis was used to verify the specific single-product amplification. The gene expression levels were normalized relative to the expression levels of the *cpxR* housekeeping gene and relative quantifications were determined with CFX Manager software (Bio-Rad) by the E(−Delta Delta C(T)). The amplification efficiency (E) of each primer pair used for the quantification was calculated from a standard amplification curve obtained by four dilution series of genomic DNA. All assays were performed in technical triplicates with three independently isolated RNA samples.

### Data analysis

Correlation between RNAseq and RT-qPCR was analyzed using Pearson’s correlation test in GraphPad Prism. Z-scores were calculated from the normalized DESeq expression data by the following formula: (X-Y)/Z (X: normalized DESeq counts of the sample; Y: average normalized DESeq counts of all the considered samples; Z: standard error of the counts mean for all the considered samples). Z-score values were used as a matrix to perform a principal component analysis and heatmaps with packages of R/Bioconductor: FactoMineR and Heatmap.2 (gplots), respectively. Column clustering was hierarchical, and two methods were used to cluster lines: hierarchical clustering and K-means clustering methods [54]. K-means clustering was applied with different values of K (i.e. the number of clusters): 1 to 13. The clearest representation for each condition of the dataset was obtained with K = 10 for CDS clustering and K = 5 for ncRNA genes clustering. To highlight groups of CDS highly overexpressed or under-expressed in a specific condition, the mean of the Z-scores in each cluster was calculated for each condition, and the Z-score groups presenting a mean value > 1 or < −1 were named overexpressed boxes and under-expressed boxes, respectively.

The most relevant signature genes in the dataset were extracted using two fold-change thresholds, the *Identity Threshold Fold-Change* and the *Differential Threshold Fold-Change*. These thresholds were modulated as described in Figure S4 (Additional file 7) to obtain the most stringent signature genes for each condition.

### Availability of supporting data

The RNA-seq data sets supporting the results of this article have been deposited in NCBI’s Gene Expression Omnibus and are accessible through GEO Series accession number GSE71754 (https://www.ncbi.nlm.nih.gov/geo/query/acc.cgi?acc=GSE71754). All the supporting data are included as Additional files.

## Additional files

Additional file 1: Movie S1. Biofilm development of *K. pneumoniae* CH1034. *K. pneumoniae* CH1034-*gfp* was cultivated in flow-cell at 37 °C with a constant flux of medium. Biofilm development and maturation were monitored by confocal microscopy. The biofilm structure evolved from a flat to a three-dimensional structure. (MPG 3450 kb) Additional file 2: Table S1. Data relative to the 2 052 selected CDS. (XLSX 974 kb) Additional file 3: Table S2. Data relative to the 19 selected ncRNA genes. (XLSX 18 kb) Additional file 4: Figure S1. Determination of the correlation index between RNAseq and RT-qPCR data. Relative expression levels of 20 randomly selected genes were determined in bacteria collected in the effluent compared to the 13 h-old biofilm. The RNAseq and RT-qPCR ratios were then log2 transformed and values were plotted against each other to evaluate their correlation. The correlation coefficient was deduced from a linear regression of the plotted values using Pearson’s correlation test in GraphPad Prism. RT-qPCRs were performed with three biological replicates of total RNA extracts. Data were normalized to the endogenous reference gene *cpxR*, whose expression did not show significant variation between the tested conditions according to the RNAseq data. (PDF 123 kb) Additional file 5: Figure S2. Representation of the transcriptomic profiles of planktonic, sessile and biofilm-dispersed cells. The heatmap represents the hierarchical clustering of the Z-score of each of the 2 052 genes differentially expressed in at least one of the 10 possible pairs of conditions. Each condition was composed of three biological replicates, which were clustered together. Columns were clustered with the hierarchical clustering. (PDF 926 kb) Additional file 6: Figure S3. Clusters of Orthologous Group (COG) affiliation of the genes of each K-means cluster. The circle size is proportional to the percentage of genes (indicated by numbers) affiliated to a COG category for one given cluster group. Percentages in bold characters correspond to the major part of each cluster. (PDF 311 kb) Additional file 7: Figure S4. Strategy used for signature gene identification. Two thresholds were used: an “*Identity Threshold Fold-Change”* and a “*Differential Threshold Fold-Change.* Their respective values are indicated below. As an example, here is presented the strategy employed to identify one signature gene of the 13 h-old biofilm condition. The absolute expression (baseMeans) of the gene is represented by a filled circle in the 13 h-old biofilm condition, and by empty circles in the other conditions. Signature gene is defined according to two characteristics: i) differential expression levels between the 13 h-old biofilm condition (filled circle) and the other conditions (empty circles) higher than 4 (*Differential Threshold Fold-Change*), and ii) differential expression levels between all other conditions (empty circles) less than 2.5 (*Identity Threshold Fold-Change*). BaseMeans correspond to the absolute expression values averaged for triplicates of a condition as calculated by the DESeq package. (PDF 147 kb) Additional file 8: Table S3. List of primers used in this study. (XLSX 12 kb)

## Abbreviations

Bp: base pair
c-di-GMP: bis-(3'-5')-cyclic dimeric Guanosine Monophosphate
CDS: Coding DNA Sequences
cDNA: complementary DNA
CFU: colony forming unit
COG: Clusters of Orthologous Groups
DEPC: diethylpyrocarbonate
GEO: Gene Expression Omnibus
GFP: green fluorescent prrotein
LB: Lysogeny broth
ncRNA: non-coding RNA
OD_620_: optical density at 620 nm
PBS: phosphate buffer saline
PCA: principal component analysis
PCR: polymerase chain reaction
PTS: phosphotransferase system
qPCR: quantitative Polymerase Chain Reaction
RNA-seq: high-throughput sequencing of RNA
rRNA: ribosomal RNA
RT-qPCR: reverse transcription-quantitative polymerase chain reaction
tRNA: transfer RNA
vol/vol: volume/volume
wt/vol: weight/volume
σ^S^: sigma S factor

## References

[1] Costerton JW, Stewart PS, Greenberg EP (1999). Bacterial Biofilms: A Common Cause of Persistent Infections. Science.

[2] Bogino PC, de las Mercedes Oliva M, Sorroche FG, Giordano W (2013). The Role of Bacterial Biofilms and Surface Components in Plant-Bacterial Associations. Int J Mol Sci.

[3] Karatan E, Watnick P (2009). Signals, Regulatory Networks, and Materials That Build and Break Bacterial Biofilms. Microbiol Mol Biol Rev.

[4] Petrova OE, Cherny KE, Sauer K (2015). The Diguanylate Cyclase GcbA Facilitates *Pseudomonas aeruginosa* Biofilm Dispersion by Activating BdlA. J Bacteriol.

[5] Beloin C, Roux A, Ghigo J-M (2008). *Escherichia coli* biofilms. Curr Top Microbiol Immunol.

[6] Flemming H-C, Wingender J (2010). The biofilm matrix. Nat Rev Microbiol.

[7] Laverty G, Gorman SP, Gilmore BF (2014). Biomolecular Mechanisms of *Pseudomonas aeruginosa* and *Escherichia coli* Biofilm Formation. Pathogens.

[8] Hall-Stoodley L, Costerton JW, Stoodley P (2004). Bacterial biofilms: from the Natural environment to infectious diseases. Nat Rev Microbiol.

[9] Otto M (2013). Staphylococcal infections: mechanisms of biofilm maturation and detachment as critical determinants of pathogenicity. Annu Rev Med.

[10] Kaplan JB (2010). Biofilm Dispersal. J Dent Res.

[11] McDougald D, Rice SA, Barraud N, Steinberg PD, Kjelleberg S (2012). Should we stay or should we go: mechanisms and ecological consequences for biofilm dispersal. Nat Rev Microbiol.

[12] Kaplan JB, Ragunath C, Ramasubbu N, Fine DH (2003). Detachment of Actinobacillus actinomycetemcomitans Biofilm Cells by an Endogenous β-Hexosaminidase Activity. J Bacteriol.

[13] Gjermansen M, Nilsson M, Yang L, Tolker-Nielsen T (2010). Characterization of starvation-induced dispersion in *Pseudomonas putida* biofilms: genetic elements and molecular mechanisms. Mol Microbiol.

[14] Cho C, Chande A, Gakhar L, Bakaletz LO, Jurcisek JA, Ketterer M, et al. Role of the Nuclease of Nontypeable *Haemophilus influenzae* in Dispersal of Organisms from Biofilms. Infect Immun. 2014. doi: 10.1128/IAI.02601-14 10.1128/IAI.02601-14 PMC4333478 25547799

[15] Wang R, Khan BA, Cheung GYC, Bach T-HL, Jameson-Lee M, Kong K-F (2011). *Staphylococcus epidermidis* surfactant peptides promote biofilm maturation and dissemination of biofilm-associated infection in mice. J Clin Invest.

[16] Periasamy S, Joo H-S, Duong AC, Bach T-HL, Tan VY, Chatterjee SS (2012). How *Staphylococcus aureus* biofilms develop their characteristic structure. Proc Natl Acad Sci U S A.

[17] Rice SA, Tan CH, Mikkelsen PJ, Kung V, Woo J, Tay M (2009). The biofilm life cycle and virulence of *Pseudomonas aeruginosa* are dependent on a filamentous prophage. ISME J.

[18] Rossmann FS, Racek T, Wobser D, Puchalka J, Rabener EM, Reiger M (2015). Phage-mediated Dispersal of Biofilm and Distribution of Bacterial Virulence Genes Is Induced by Quorum Sensing. PLoS Pathog.

[19] Beloin C, Valle J, Latour-Lambert P, Faure P, Kzreminski M, Balestrino D (2004). Global impact of mature biofilm lifestyle on *Escherichia coli* K-12 gene expression. Mol Microbiol.

[20] Dötsch A, Eckweiler D, Schniederjans M, Zimmermann A, Jensen V, Scharfe M (2012). The *Pseudomonas aeruginosa* Transcriptome in Planktonic Cultures and Static Biofilms Using RNA Sequencing. PLoS ONE.

[21] Rumbo-Feal S, Gómez MJ, Gayoso C, Alvarez-Fraga L, Cabral MP, Aransay AM (2013). Whole transcriptome analysis of *Acinetobacter baumannii* assessed by RNA-sequencing reveals different mRNA expression profiles in biofilm compared to planktonic cells. PLoS One.

[22] Balestrino D, Ghigo J-M, Charbonnel N, Haagensen JAJ, Forestier C (2008). The characterization of functions involved in the establishment and maturation of *Klebsiella pneumoniae* in vitro biofilm reveals dual roles for surface exopolysaccharides. Environ Microbiol.

[23] Schroll C, Barken KB, Krogfelt KA, Struve C (2010). Role of type 1 and type 3 fimbriae in *Klebsiella pneumoniae* biofilm formation. BMC Microbiol.

[24] Chua SL, Liu Y, Yam JKH, Chen Y, Vejborg RM, Tan BGC (2014). Dispersed cells represent a distinct stage in the transition from bacterial biofilm to planktonic lifestyles. Nat Commun.

[25] Chua SL, Hultqvist LD, Yuan M, Rybtke M, Nielsen TE, Givskov M (2015). In vitro and in vivo generation and characterization of *Pseudomonas aeruginosa* biofilm-dispersed cells via c-di-GMP manipulation. Nat Protoc.

[26] Lange R, Hengge-Aronis R (1991). Identification of a central regulator of stationary-phase gene expression in *Escherichia coli*. Mol Microbiol.

[27] Dong T, Schellhorn HE (2009). Global effect of RpoS on gene expression in pathogenic *Escherichia coli* O157:H7 strain EDL933. BMC Genomics.

[28] Borisov VB, Gennis RB, Hemp J, Verkhovsky MI (2011). The cytochrome bd respiratory oxygen reductases. Biochim Biophys Acta.

[29] VanOrsdel CE, Bhatt S, Allen RJ, Brenner EP, Hobson JJ, Jamil A (2013). The *Escherichia coli* CydX Protein Is a Member of the CydAB Cytochrome bd Oxidase Complex and Is Required for Cytochrome bd Oxidase Activity. J Bacteriol.

[30] Mika F, Hengge R (2014). Small RNAs in the control of RpoS, CsgD, and biofilm architecture of *Escherichia coli*. RNA Biol.

[31] MacKenzie KD, Wang Y, Shivak DJ, Wong CS, Hoffman LJL, Lam S (2015). Bistable Expression of CsgD in *Salmonella enterica* Serovar Typhimurium Connects Virulence to Persistence. Infect Immun.

[32] Waite RD, Paccanaro A, Papakonstantinopoulou A, Hurst JM, Saqi M, Littler E (2006). Clustering of *Pseudomonas aeruginosa* transcriptomes from planktonic cultures, developing and mature biofilms reveals distinct expression profiles. BMC Genomics.

[33] Valle J, Da Re S, Schmid S, Skurnik D, D’Ari R, Ghigo J-M (2008). The Amino Acid Valine Is Secreted in Continuous-Flow Bacterial Biofilms. J Bacteriol.

[34] Hamilton S, Bongaerts RJ, Mulholland F, Cochrane B, Porter J, Lucchini S (2009). The transcriptional programme of *Salmonella enterica* serovar Typhimurium reveals a key role for tryptophan metabolism in biofilms. BMC Genomics.

[35] Domka J, Lee J, Wood TK (2006). YliH (BssR) and YceP (BssS) Regulate *Escherichia coli* K-12 Biofilm Formation by Influencing Cell Signaling. Appl Environ Microbiol.

[36] Kuczyńska-Wiśnik D, Matuszewska E, Laskowska E (2010). *Escherichia coli* heat-shock proteins IbpA and IbpB affect biofilm formation by influencing the level of extracellular indole. Microbiol Read Engl.

[37] Solano C, Echeverz M, Lasa I (2014). Biofilm dispersion and quorum sensing. Curr Opin Microbiol.

[38] Pereira CS, Thompson JA, Xavier KB (2013). AI-2-mediated signalling in bacteria. FEMS Microbiol Rev.

[39] Webb JS, Thompson LS, James S, Charlton T, Tolker-Nielsen T, Koch B (2003). Cell Death in *Pseudomonas aeruginosa* Biofilm Development. J Bacteriol.

[40] Brissette JL, Russel M, Weiner L, Model P (1990). Phage shock protein, a stress protein of *Escherichia coli*. Proc Natl Acad Sci U S A.

[41] Darwin AJ (2013). Stress Relief during Host Infection: The Phage Shock Protein Response Supports Bacterial Virulence in Various Ways. PLoS Pathog.

[42] Roy AB, Petrova OE, Sauer K (2012). The Phosphodiesterase DipA (PA5017) Is Essential for *Pseudomonas aeruginosa* Biofilm Dispersion. J Bacteriol.

[43] Wilksch JJ, Yang J, Clements A, Gabbe JL, Short KR, Cao H (2011). MrkH, a Novel c-di-GMP-Dependent Transcriptional Activator, Controls *Klebsiella pneumoniae* Biofilm Formation by Regulating Type 3 Fimbriae Expression. PLoS Pathog.

[44] Barraud N, Hassett DJ, Hwang S-H, Rice SA, Kjelleberg S, Webb JS (2006). Involvement of Nitric Oxide in Biofilm Dispersal of *Pseudomonas aeruginosa*. J Bacteriol.

[45] Takada H, Morita M, Shiwa Y, Sugimoto R, Suzuki S, Kawamura F (2014). Cell motility and biofilm formation in *Bacillus subtilis* are affected by the ribosomal proteins, S11 and S21. Biosci Biotechnol Biochem.

[46] Chacón KN, Mealman TD, McEvoy MM, Blackburn NJ (2014). Tracking metal ions through a Cu/Ag efflux pump assigns the functional roles of the periplasmic proteins. Proc Natl Acad Sci U S A.

[47] Guilhen C, Taha M-K, Veyrier FJ (2013). Role of transition metal exporters in virulence: the example of *Neisseria meningitidis*. Front Cell Infect Microbiol.

[48] Yan Y, Su S, Meng X, Ji X, Qu Y, Liu Z (2013). Determination of sRNA Expressions by RNA-seq in *Yersinia pestis* Grown In Vitro and during Infection. PLoS ONE.

[49] Ortega ÁD, Gonzalo-Asensio J, Portillo FG (2012). Dynamics of *Salmonella* small RNA expression in non-growing bacteria located inside eukaryotic cells. RNA Biol.

[50] Guilhen C, Iltis A, Forestier C, Balestrino D. Genome Sequence of a Clinical *Klebsiella pneumoniae* Sequence Type 6 Strain. Genome Announc. 2015;3(6):e01311-5. 10.1128/genomeA.01311-15 PMC4972775 26564039

[51] Weiss Nielsen M, Sternberg C, Molin S, Regenberg B. *Pseudomonas aeruginosa* and *Saccharomyces cerevisiae* Biofilm in Flow Cells. J Vis Exp JoVE. 2011;(47):e2383. 10.3791/2383 PMC3182659 21304454

[52] Toledo-Arana A, Dussurget O, Nikitas G, Sesto N, Guet-Revillet H, Balestrino D (2009). The *Listeria* transcriptional landscape from saprophytism to virulence. Nature.

[53] Li H, Durbin R (2009). Fast and accurate short read alignment with Burrows–Wheeler transform. Bioinformatics.

[54] Sherlock G (2000). Analysis of large-scale gene expression data. Curr Opin Immunol.

