# Supplementary figures and images for: Transcriptional profiling of Klebsiella pneumoniae defines signatures for planktonic, sessile and biofilm-dispersed cells
Source: BMC Genomics. 2016 Mar 15;17:237. doi: 10.1186/s12864-016-2557-x (PMC4791964; doi:10.1186/s12864-016-2557-x)

## Additional file 4: Figure S1

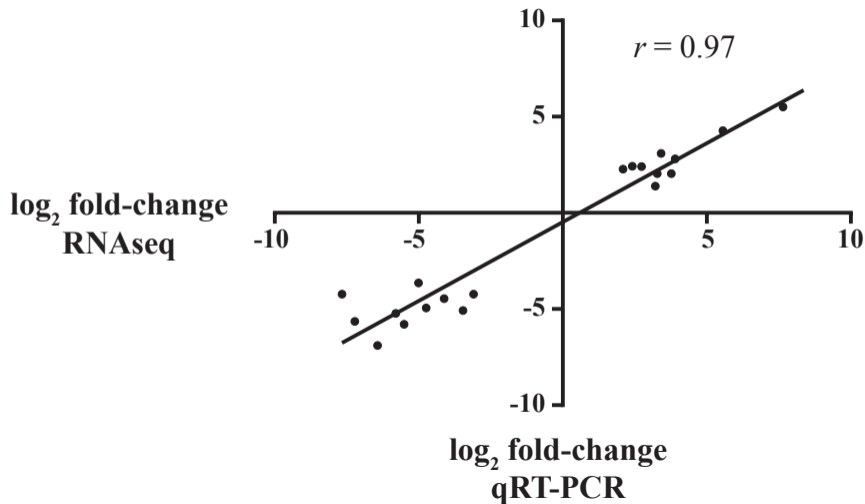

Supplement: Additional file 4: Figure S1. — Determination of the correlation index between RNAseq and RT-qPCR data. Relative expression levels of 20 randomly selected genes were determined in bacteria collected in the effluent compared to the 13 h-old biofilm. The RNAseq and RT-qPCR ratios were then log2 transformed and values were plotted against each other to evaluate their correlation. The correlation coefficient was deduced from a linear regression of the plotted values using Pearson’s correlation test in GraphPad Prism. RT-qPCRs were performed with three biological replicates of total RNA extracts. Data were normalized to the endogenous reference gene cpxR, whose expression did not show significant variation between the tested conditions according to the RNAseq data. (PDF 123 kb) [file 12864_2016_2557_MOESM4_ESM.pdf]

**Additional file 5: Figure S2**

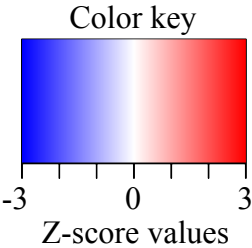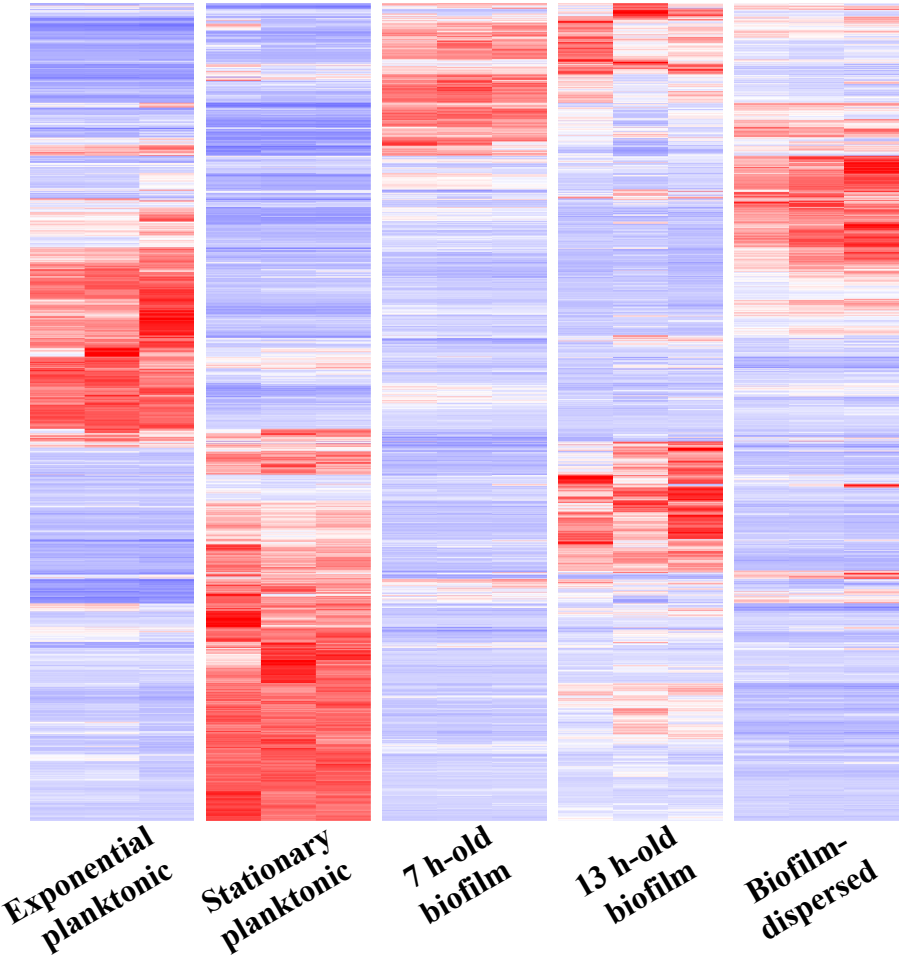

Supplement: Additional file 5: Figure S2. — Representation of the transcriptomic profiles of planktonic, sessile and biofilm-dispersed cells. The heatmap represents the hierarchical clustering of the Z-score of each of the 2 052 genes differentially expressed in at least one of the 10 possible pairs of conditions. Each condition was composed of three biological replicates, which were clustered together. Columns were clustered with the hierarchical clustering. (PDF 926 kb) [file 12864_2016_2557_MOESM5_ESM.pdf]

Additional file 6: Figure S3

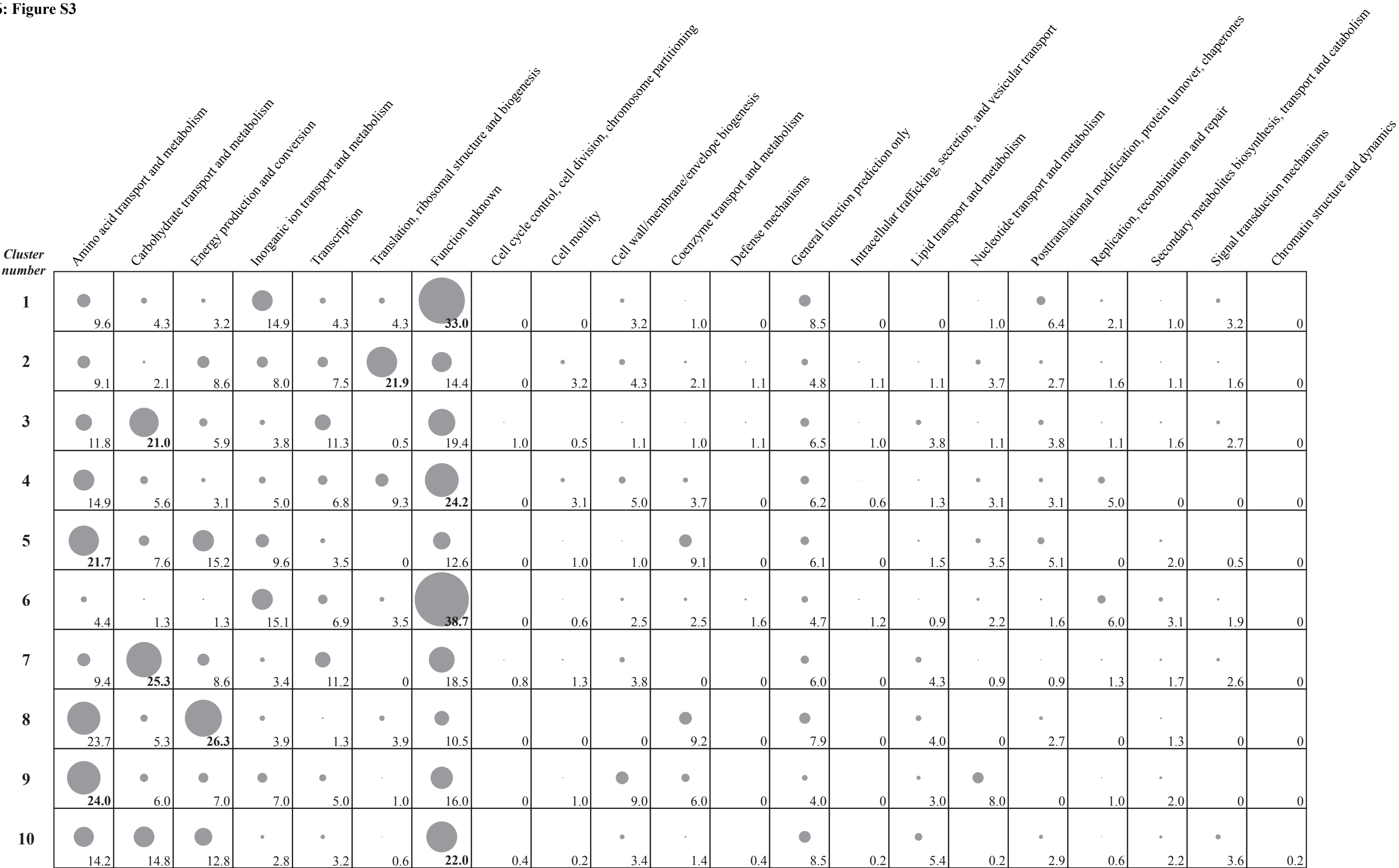

Supplement: Additional file 6: Figure S3. — Clusters of Orthologous Group (COG) affiliation of the genes of each K-means cluster. The circle size is proportional to the percentage of genes (indicated by numbers) affiliated to a COG category for one given cluster group. Percentages in bold characters correspond to the major part of each cluster. (PDF 311 kb) [file 12864_2016_2557_MOESM6_ESM.pdf]

Additional file 7: Figure S4

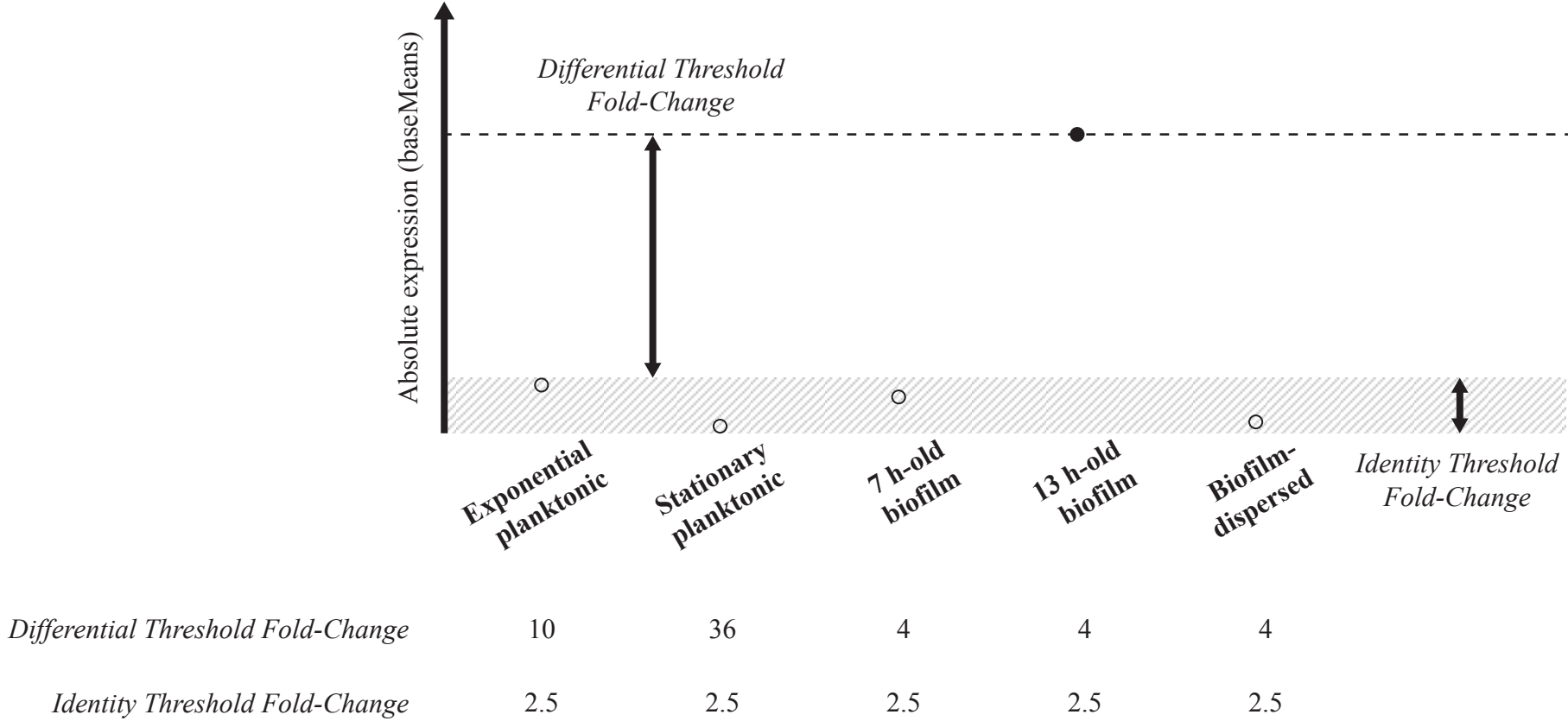

Supplement: Additional file 7: Figure S4. — Strategy used for signature gene identification. Two thresholds were used: an “Identity Threshold Fold-Change” and a “Differential Threshold Fold-Change. Their respective values are indicated below. As an example, here is presented the strategy employed to identify one signature gene of the 13 h-old biofilm condition. The absolute expression (baseMeans) of the gene is represented by a filled circle in the 13 h-old biofilm condition, and by empty circles in the other conditions. Signature gene is defined according to two characteristics: i) differential expression levels between the 13 h-old biofilm condition (filled circle) and the other conditions (empty circles) higher than 4 (Differential Threshold Fold-Change), and ii) differential expression levels between all other conditions (empty circles) less than 2.5 (Identity Threshold Fold-Change). BaseMeans correspond to the absolute expression values averaged for triplicates of a condition as calculated by the DESeq package. (PDF 147 kb) [file 12864_2016_2557_MOESM7_ESM.pdf]
